# Supplementary material for: Getting More Out of Biomedical Documents with GATE's Full Lifecycle Open Source Text Analytics
Source: PLoS Comput Biol. 2013 Feb 7;9(2):e1002854. doi: 10.1371/journal.pcbi.1002854 (PMC3567135; doi:10.1371/journal.pcbi.1002854)
Supplement: Dataset S1 — GATE software. Dataset S1 bundled with this paper contains a distribution of GATE (or see http://gate.ac.uk/download/). (TGZ) [file pcbi.1002854.s001.tgz › plos-gate/src/gate/resources/gate.ac.uk/tests/html/test1.htm]

Relativity: Appendix III


Albert Einstein: Relativity
  
Appendix

---

### Appendix III

## The Experimental Confirmation of the General Theory of Relativity

---

From a systematic theoretical point of view, we may imagine the process of evolution of an empirical science to be a continuous process of induction. Theories are evolved and are expressed in short compass as statements of a large number of individual observations in the form of empirical laws, from which the general laws can be ascertained by comparison. Regarded in this way, the development of a science bears some resemblance to the compilation of a classified catalogue. It is, as it were, a purely empirical enterprise.

But this point of view by no means embraces the whole of the actual process ; for it slurs over the important part played by intuition and deductive thought in the development of an exact science. As soon as a science has emerged from its initial stages, theoretical advances are no longer achieved merely by a process of arrangement. Guided by empirical data, the investigator rather develops a system of thought which, in general, is built up logically from a small number of fundamental assumptions, the so-called axioms. We call such a system of thought a *theory.* The theory finds the justification for its existence in the fact that it correlates a large number of single observations, and it is just here that the " truth " of the theory lies.

Corresponding to the same complex of empirical data, there may be several theories, which differ from one another to a considerable extent. But as regards the deductions from the theories which are capable of being tested, the agreement between the theories may be so complete that it becomes difficult to find any deductions in which the two theories differ from each other. As an example, a case of general interest is available in the province of biology, in the Darwinian theory of the development of species by selection in the struggle for existence, and in the theory of development which is based on the hypothesis of the hereditary transmission of acquired characters.

We have another instance of far-reaching agreement between the deductions from two theories in Newtonian mechanics on the one hand, and the general theory of relativity on the other. This agreement goes so far, that up to the preseat we have been able to find only a few deductions from the general theory of relativity which are capable of investigation, and to which the physics of pre-relativity days does not also lead, and this despite the profound difference in the fundamental assumptions of the two theories. In what follows, we shall again consider these important deductions, and we shall also discuss the empirical evidence appertaining to them which has hitherto been obtained.

#### (a) Motion of the Perihelion of Mercury

According to Newtonian mechanics and Newton's law of gravitation, a planet which is revolving round the sun would describe an ellipse round the latter, or, more correctly, round the common centre of gravity of the sun and the planet. In such a system, the sun, or the common centre of gravity, lies in one of the foci of the orbital ellipse in such a manner that, in the course of a planet-year, the distance sun-planet grows from a minimum to a maximum, and then decreases again to a minimum. If instead of Newton's law we insert a somewhat different law of attraction into the calculation, we find that, according to this new law, the motion would still take place in such a manner that the distance sun-planet exhibits periodic variations; but in this case the angle described by the line joining sun and planet during such a period (from perihelion—closest proximity to the sun—to perihelion) would differ from 360^0^. The line of the orbit would not then be a closed one but in the course of time it would fill up an annular part of the orbital plane, viz. between the circle of least and the circle of greatest distance of the planet from the sun.

According also to the general theory of relativity, which differs of course from the theory of Newton, a small variation from the Newton-Kepler motion of a planet in its orbit should take place, and in such away, that the angle described by the radius sun-planet between one perhelion and the next should exceed that corresponding to one complete revolution by an amount given by


(*N.B.* — One complete revolution corresponds to the angle 2π in the absolute angular measure customary in physics, and the above expression giver the amount by which the radius sun-planet exceeds this angle during the interval between one perihelion and the next.) In this expression a represents the major semi-axis of the ellipse, e its eccentricity, c the velocity of light, and T the period of revolution of the planet. Our result may also be stated as follows : According to the general theory of relativity, the major axis of the ellipse rotates round the sun in the same sense as the orbital motion of the planet. Theory requires that this rotation should amount to 43 seconds of arc per century for the planet Mercury, but for the other Planets of our solar system its magnitude should be so small that it would necessarily escape detection. ^1)^

In point of fact, astronomers have found that the theory of Newton does not suffice to calculate the observed motion of Mercury with an exactness corresponding to that of the delicacy of observation attainable at the present time. After taking account of all the disturbing influences exerted on Mercury by the remaining planets, it was found (Leverrier: 1859; and Newcomb: 1895) that an unexplained perihelial movement of the orbit of Mercury remained over, the amount of which does not differ sensibly from the above mentioned +43 seconds of arc per century. The uncertainty of the empirical result amounts to a few seconds only.

#### (b) Deflection of Light by a Gravitational Field

In Section 22 it has been already mentioned that according to the general theory of relativity, a ray of light will experience a curvature of its path when passing through a gravitational field, this curvature being similar to that experienced by the path of a body which is projected through a gravitational field. As a result of this theory, we should expect that a ray of light which is passing close to a heavenly body would be deviated towards the latter. For a ray of light which passes the sun at a distance of Δ sun-radii from its centre, the angle of deflection (a) should amount to


It may be added that, according to the theory, half of  this deflection is produced by the Newtonian field of attraction of the sun, and the other half by the geometrical modification (" curvature ") of space caused by the sun.

This result admits of an experimental test by means of the photographic registration of stars during a total eclipse of the sun. The only reason why we must wait for a total eclipse is because at every other time the atmosphere is so strongly illuminated by the light from the sun that the stars situated near the sun's disc are invisible. The predicted effect can be
seen clearly from the accompanying diagram. If the
sun (S) were not present, a star which is practically
infinitely distant would be seen in the direction D_1_, as
observed front the earth. But as a consequence of the deflection of light from the star by the sun, the star will be seen in the direction D_2_, *i.e.* at a somewhat greater distance from the centre of the sun than corresponds to its real position.

In practice, the question is tested in the following way. The stars in the neighbourhood of the sun are photographed during a solar eclipse. In addition, a second photograph of the same stars is taken when the sun is situated at another position in the sky, *i.e.* a few months earlier or later. As compared whh the standard photograph, the positions of the stars on the eclipse-photograph ought to appear displaced radially outwards (away from the centre of the sun) by an amount corresponding to the angle a.

We are indebted to the [British] Royal Society and to the Royal Astronomical Society for the investigation of this important deduction. Undaunted by the [first world] war and by difficulties of both a material and a psychological nature aroused by the war, these societies equipped two expeditions — to Sobral (Brazil), and to the island of Principe (West Africa) — and sent several of Britain's most celebrated astronomers (Eddington, Cottingham, Crommelin, Davidson), in order to obtain photographs of the solar eclipse of 29th May, 1919. The relative discrepancies to be expected between the stellar photographs obtained during the eclipse and the comparison photographs amounted to a few hundredths of a millimetre only. Thus great accuracy was necessary in making the adjustments required for the taking of the photographs, and in their subsequent measurement.

The results of the measurements confirmed the theory in a thoroughly satisfactory manner. The rectangular components of the observed and of the calculated deviations of the stars (in seconds of arc) are set forth in the following table of results :


#### (c) Displacement of Spectral Lines Towards the Red

In Section 23 it has been shown that in a system K^1^ which is in rotation with regard to a Galileian system K, clocks of identical construction, and which are considered at rest with respect to the rotating reference-body, go at rates which are dependent on the positions of the clocks. We shall now examine this dependence quantitatively. A clock, which is situated at a distance r from the centre of the disc, has a velocity relative to K which is given by

V = wr

where w represents the angular velocity of rotation of the disc K^1^ with respect to K. If v_0_, represents the number of ticks of the clock per unit time (" rate " of the clock) relative to K when the clock is at rest, then the " rate " of the clock (v) when it is moving relative to K with a velocity V, but at rest with respect to the disc, will, in accordance with Section 12, be given by


or with sufficient accuracy by


This expression may also be stated in the following form:


If we represent the difference of potential of the centrifugal force between the position of the clock and the centre of the disc by φ, *i.e.* the work, considered negatively, which must be performed on the unit of mass against the centrifugal force in order to transport it from the position of the clock on the rotating disc to the centre of the disc, then we have


From this it follows that


In the first place, we see from this expression that two clocks of identical construction will go at different rates when situated at different distances from the centre of the disc. This result is aiso valid from the standpoint of an observer who is rotating with the disc.

Now, as judged from the disc, the latter is in a gravititional field of potential φ, hence the result we have obtained will hold quite generally for gravitational fields. Furthermore, we can regard an atom which is emitting spectral lines as a clock, so that the following statement will hold:

*An atom absorbs or emits light of a frequency which is dependent on the potential of the gravitational field in which it is situated.*

The frequency of an atom situated on the surface of a heavenly body will be somewhat less than the frequency of an atom of the same element which is situated in free space (or on the surface of a smaller celestial body).

Now φ = - K (M/r), where K is Newton's constant of gravitation, and M is the mass of the heavenly body. Thus a displacement towards the red ought to take place for spectral lines produced at the surface of stars as compared with the spectral lines of the same element produced at the surface of the earth, the amount of this displacement being


For the sun, the displacement towards the red predicted by theory amounts to about two millionths of the wave-length. A trustworthy calculation is not possible in the case of the stars, because in general neither the mass M nor the radius r are known.

It is an open question whether or not this effect exists, and at the present time (1920) astronomers are working with great zeal towards the solution. Owing to the smallness of the effect in the case of the sun, it is difficult to form an opinion as to its existence. Whereas Grebe and Bachem (Bonn), as a result of their own measurements and those of Evershed and Schwarzschild on the cyanogen bands, have placed the existence of the effect almost beyond doubt, while other investigators, particularly St. John, have been led to the opposite opinion in consequence of their measurements.

Mean displacements of lines towards the less refrangible end of the spectrum are certainly revealed by statistical investigations of the fixed stars ; but up to the present the examination of the available data does not allow of any definite decision being arrived at, as to whether or not these displacements are to be referred in reality to the effect of gravitation. The results of observation have been collected together, and discussed in detail from the standpoint of the question which has been engaging our attention here, in a paper by E. Freundlich entitled "Zur Prüfung der allgemeinen Relativitâts-Theorie" (*Die Naturwissenschaften,* 1919, No. 35, p. 520: Julius Springer, Berlin).

At all events, a definite decision will be reached during the next few years. If the displacement of spectral lines towards the red by the gravitational potential does not exist, then the general theory of relativity will be untenable. On the other hand, if the cause of the displacement of spectral lines be definitely traced to the gravitational potential, then the study of this displacement will furnish us with important information as to the mass of the heavenly bodies. ^[A]^

Next: Appendix IV: The Structure of Space According to the General Theory of Relativity

---

#### Footnotes

^1)^
Especially since the next planet Venus has an orbit that is almost an exact circle, which makes it more difficult to locate the perihelion with precision.

^[A]^
The displacentent of spectral lines towards the red end of the spectrum was definitely established by Adams in 1924, by observations on the dense companion of Sirius, for which the effect is about thirty times greater than for the Sun. *R.W.L. — translator*

---

Relativity: The Special and General Theory
